# Sugarcane Bagasse-Derived Cellulose Nanocrystal/Polyvinyl Alcohol/Gum Tragacanth Composite Film Incorporated with Betel Leaf Extract as a Versatile Biomaterial for Wound Dressing

**DOI:** 10.1155/2023/9630168

**Published:** 2023-07-13

**Authors:** Luong Ngoc Diem, Selorm Torgbo, Indranil Banerjee, Kunal Pal, Udomlak Sukatta, Prapassorn Rugthaworn, Prakit Sukyai

**Affiliations:** ^1^Cellulose for Future Materials and Technologies Special Research Unit, Department of Biotechnology, Faculty of Agro-Industry, Kasetsart University, Chatuchak, Bangkok 10900, Thailand; ^2^Department of Bioscience and Bioengineering, Indian Institute of Technology Jodhpur, NH-65 Karwar, Jodhpur 342037, India; ^3^Department of Biotechnology and Medical Engineering, National Institute of Technology, Rourkela, Odisha 769008, India; ^4^Kasetsart Agriculture and Agro-Industrial Product Improvement Institute, Kasetsart University, Bangkok 10900, Thailand; ^5^Center for Advanced Studies for Agriculture and Food (CASAF), Kasetsart University Institute for Advanced Studies, Kasetsart University, Chatuchak, Bangkok 10900, Thailand

## Abstract

In this study, nanocomposite film was fabricated using cellulose nanocrystals (CNCs) as nanofiller in a polymer matrix of polyvinyl alcohol (PVA) and gum tragacanth (GT) via solution casting. CNCs were extracted from sugarcane bagasse using a steam explosion technique followed by acid hydrolysis. Initial analysis of CNCs by transmission electron microscopy (TEM) showed nanosized particles of 104 nm in length and 7 nm in width. Physical and chemical characteristics of neat PVA, PVA/GT, and PVA/GT/CNC films with varying concentrations of CNCs (from 2% to 10%) were analyzed by the scanning electron microscopy (SEM), Fourier transform infrared (FTIR) spectrometry, mechanical test, and swelling test. The SEM analysis showed cluster formation of CNCs in the polymer matrix at high concentration. The developed films were transparent. FTIR spectrometry analysis confirmed the chemical functional groups of the various components in the film. The presence of GT and CNCs in the polymer matrix improved the characteristics of films as evident in the prolonged stability for 7 days and increased mechanical properties. The highest elastic modulus of 1526.11 ± 31.86 MPa and tensile strength of 80.39 MPa were recorded in PVA/GT/CNC2 film. The swelling ability, however, decreased from 260% to 230%. Cytotoxicity analysis of the PVA/GT/CNC film showed that it is nontoxic to mouse fibroblast cells L929 with 95% cell viability. Films loaded with betel leaf extract exhibited excellent antibacterial activities against *Staphylococcus aureus* DMST 8840 and *Pseudomonas aeruginosa* TISTR 781 with 28.20 ± 0.84 mm and 23.60 ± 0.55 mm inhibition zones, respectively. These results demonstrate that PVA/GT/CNC loaded with the betel leaf extract could act as promising and versatile wound dressings to protect the wound surface from infection and dehydration.

## 1. Introduction

Natural and synthetic polymers have been investigated over the years as biomaterials for biomedical applications [1, 2]. To blend natural polymers with synthetic ones is another unique way of preparing versatile polymeric materials for biomedical applications [3]. The synthetic polymers have good mechanical properties and thermal stability; however, they can inhibit cell growth due to residue of initiators and other compounds. Natural polymers on the other hand are usually biodegradable, biocompatible, and bioactive in nature [3–5]. Polyvinyl alcohol (PVA) is a linear synthetic polymer which is nontoxic, biocompatible, thermostable, and water-soluble with film forming properties [6, 7]. PVA has been used as the main matrix with outstanding physical and chemical properties in biomedical applications [8, 9]. However, the scope of PVA application is limited by its instability, insufficient elasticity, and lack of cell-specific bioactivities [9, 10]. To tackle these problems, several attempts have been made by incorporating substances such as chitosan [11, 12], dextran [13], polyion liquid [14], and nanocellulose [15, 16]. PVA-based composite films containing bacterial cellulose and epsilon polylysine [16], dextran aldehyde hydrogel [13], and silk fibroin-PVA composite film coated with chitosan-ZnO nanoparticles [17] were reported for wound dressings. These dressings demonstrated good absorbability and tissue regeneration with low cytotoxicity and inhibited bacterial infections with enhanced mechanical properties. However, these materials lacked essential features such as good swelling properties and mediated angiogenesis.

Gum tragacanth (GT) comprises a complex mixture of highly branched heterogeneous hydrophilic polysaccharides mainly D-galacturonic acid and other sugar units with numerous functional groups that provide a suitable medium for cell growth and mediated angiogenesis [18–20]. GT is one of the most abundant and renewable natural raw materials. GT is readily accessible, relatively affordable, nontoxic, biocompatible, environmentally friendly, and widely used in biomedical science [21]. As a natural polysaccharide, GT has been applied in various biotechnological industries [19]. GT has been incorporated into PVA for application in drug delivery [22, 23], scaffold for skin substitutes [24], and wound dressing [25–27]. High degradation ability, stability against microbial, and heat in the living systems have triggered several studies on GT for wound dressings [21]. A bio-based wound dressing made with gelatin, gum Arabic, and polyurethane was also reported for wound care application [28]. In a related study, PVA and Iranian gum tragacanth (IGT) biocomposite with nanoclay was reported to have superior degradation and higher chemical and mechanical stability for wound dressing [29]. In addition, GT also serves as a suitable stabilizing agent for biosynthesis of nanoparticles [30]. The major limitation of GT in biomedical application has to do with its rapid degradation, which affects the mechanical and biological properties. Interestingly, this drawback can be overcome using nanofillers such as cellulose nanocrystals.

Large amount of waste is unfortunately generated from sugarcane processing industries, which composed mainly of bagasse, molasses, filter cake, and leaves [31–33]. The bagasse and leaves are rich in cellulose fibers for nanocellulose production. Cellulose nanocrystals (CNCs) are potential reinforced nanofillers with needle-like shapes ranging from 100 nm to 250 nm in length and from 5 nm to 70 nm in width, high aspect ratio, and high crystallinity [34]. Dispersion of CNCs in synthetic polymers will provide good mechanical properties due to their unique crystalline domains with inherent stability that contributes stiffness and elasticity to the material [35]. CNCs have zero or low toxicity and can stimulate long-term cell proliferation [36]. Hence, CNCs have been applied in various aspects of medical fields [37]. The addition of CNC into carboxymethyl cellulose-glycerol (CMC-G) matrix was reported to improve the physiochemical properties and stability of the film [38]. A study on bioactive wound dressing using hyaluronic acid- (HA-) based nanofibers with PVA revealed that incorporating CNCs into PVA/HA significantly improved swelling and mechanical properties [39]. However, it lacks antimicrobial properties. Antimicrobial activity is an important factor for considering biomaterials for wound dressings. This prevents contamination or bacterial infection while improving the healing process. For that matter, plant-based bioactive compounds are widely used as an antibacterial agent in wound dressings [40, 41]. An alternative nanocomposite film for traditional wound dressing incorporated with black pepper and ginger essential oils was reported to significantly inhibit the growth of bacteria [42]. In a related study, papain immobilized in bacterial cellulose as a polymer template was reported for wound dressing with strong antibacterial action [43]. Plant extractive from betel leaf (*Piper betle* L.) was used in this study. Betel leaf is a traditional herbal medicine commonly found in Southeast Asia and East African countries that contains phenolics, flavonoids, alcohols, alkaloids, terpenes, fatty acids, and organic acids [44, 45]. These compounds were reported to exhibit several biological properties including antibacterial, antifungal, anti-inflammatory, and antioxidant activities [45–48].

Different treatment strategies and dressings with special properties are required to regenerate damaged tissues in wound management. Wound dressing material should fully cover the affected area, create conducive environment to prevent contamination, and have the ability to absorb exudates on the wound surface. In addition, it must degrade rapidly, be easy to change without causing pain or trauma at the wound bed, be nontoxic, and be cost effective [49, 50]. Film dressings are mostly preferred for superficial injuries to protect skin prone to abrasion or external contamination [50]. The transparency of film allows daily observation without its removal and prevents damage to the wound bed [49]. Therefore, combination of different polymers impregnated with the plant extract could act as novel film with unique properties for biomedical consideration in wound dressings.

Herein, PVA, GT, and CNCs were used to produce a versatile film for wound dressing. The physicochemical characteristics of the composite films which are essential for wound dressing were evaluated. Biological properties of PVA/GT/CNC were examined for the film's ability to support cell viability. The ability of the film to prevent bacterial infection was examined by loading the crude betel leaf extract as an antimicrobial agent. A preprint has previously been published [51].

## 2. Materials and Methods

### 2.1. Materials

Sugarcane bagasse was obtained from Mitr Phol Sugar Corporation., Ltd. (Klongteoy, Thailand). The chemical reagents including sodium chlorite (NaClO_2_), glacial acetic acid (CH_3_COOH), and sulfuric acid 98% (H_2_SO_4_) were purchased from Ajax Finechem Pty., Ltd. (New Zealand), Merck (Darmstadt, Germany), and QReC (New Zealand), respectively. Acetone (C_3_H_6_O) was supplied by RCL Labscan Limited (Bangkok, Thailand). Polyvinyl alcohol (PVA) (Mw = 89,000–98,000) from EMD Millipore Corporation (Germany). All other reagents used were of analytical grade.

### 2.2. Nanocellulose Extraction from Sugarcane Bagasse (SCB)

The SCB was dried at 55°C for 24 h and then treated by steam explosion (Nitto Koatsu, Japan) at 190°C with a pressure of 13 MPa for 15 min [52]. The exploded sample without sugar-rich liquid fraction was bleached by treating with 1.4% w/w sodium chlorite (NaClO_2_) solution, which was adjusted to pH of 4.0 with glacial acetic acid (CH_3_COOH) at 70°C. Bleaching chemicals were added every hour until the sample turned white. The bleached sample was then filtered and washed several times until the pH was neutral [53, 54]. The dried bleached cellulose fibers were dispersed in 64% w/v H_2_SO_4_ at a solid-to-liquid ratio of 1 : 20 with constant stirring at 500 rpm for 75 min at 45°C. The hydrolysis reaction was stopped using cold deionized (DI) water and centrifugation was repeated at 15,000 rpm for 15 min at 4°C. The supernatant was removed and replaced by clean DI water, followed by dialysis to obtain CNCs. The CNC was sonicated (Bransonic Model 2201R-MT, USA) and kept at 4°C for further use.

### 2.3. Preparation of Composite Films

An aqueous solution of PVA (10% w/v) was prepared under continuous stirring at 80°C and, 10% w/w of GT based on a specific amount of PVA was added to create PVA/GT solution [10]. The CNC composite film was fabricated by adding CNC suspension with varying concentrations (2% to 10%) to PVA/GT solution, denoted as PVA/GT/CNC2 to PVA/GT/CNC10. The mixture was stirred for 30 min to obtain a homogenous state before pouring into Petri dishes and dried at 37°C for 48 h. The dried films were soaked in crude betel leaf extract prepared in propylene glycol (PG) at different concentrations (2%, 3%, and 4% w/v) for 24 h and dried at 37°C as an antimicrobial agent.

### 2.4. Transmission Electron Microscopy (TEM)

Dimension of CNC particles was examined by TEM (Hitachi Model HT7700, Japan). Briefly, 0.01% w/v CNC suspension was deposited on a carbon-coated copper grid, poststained with 2% w/v uranyl acetate solution and dried for 8 min. The sample was analyzed with an accelerating voltage of 100 kV [55]. CNC dimensions were determined using the ImageJ program.

### 2.5. Optical and Transparency Measurement

Film transparency was determined using a Genesys 10 S UV-Vis spectrophotometer (Thermo Fisher Scientific, USA) at a wavelength of 560 nm [56]. Film specimens were cut into rectangular shapes (2 × 40 mm) and placed inside the spectrophotometer cells. An empty spectrophotometer cell was set as a blank. All measurements were conducted in triplicate and the percentage of film transparency was calculated using the following equation [57]:

(1) $$\begin{array}{c} percentage\;transparency\;\left(\%T\right)=\left(\frac{T_{f}}{T_{b}}\right)\;x\;100\text{,} \end{array}$$

where *T*_*f*_ and *T*_*b*_ are the transmittance values of the film sample and blank cell, respectively.

### 2.6. Scanning Electron Microscopy (SEM)

Surface and cross section of the composite films were investigated by SEM (Hitachi Model, Joel JSM5600LV, Japan) with an accelerating voltage of 15–20 kV [31]. To observe the cross section, film specimens were freeze-cracked following immersion in liquid nitrogen. Each piece was deposited on a cylindrical holder and coated with a thin gold layer (5–10 nm thickness) before observation.

### 2.7. Fourier Transform Infrared (FTIR) Spectrometry Analysis

The PVA/GT/CNC composite films were cut into 10 × 10 mm squares at random locations. Infrared spectra of each sample were recorded using an FTIR (Bruker Tensor 27 Spectrometer, USA) in the range of 4000 − 500 cm^−1^ with a resolution of 4 cm^−1^ in attenuated total reflection (ATR) mode.

### 2.8. Swelling Ratio and Stability

Swelling ratio and stability of the composite films were evaluated based on the amount of water absorbed and percentage of weight loss in 7 days, respectively. Briefly, dried film specimens (15 × 15 mm) were immersed in DI water at ambient temperature. For each turn of measurement [58], swelling ratio was assessed by taking the weight of swollen samples. To examine the stability of composite films, the swollen samples were taken and dried again to determine the difference in dried weight before and after immersion. All samples were weighed by an analytical balance in triplicate. The results were calculated using the following equations [13]:

(2) $$\begin{array}{c} swelling\;ratio\;\left(\%\right)=\frac{\left(M_{1}\;–\;M_{0}\right)}{M_{0}}\;x\;100\text{,} \end{array}$$

(3) $$\begin{array}{c} weight\;loss\;\left(\%\right)=\frac{\left(M_{0}\;–\;M_{1}\right)}{M_{0}}\;x\;100\text{,} \end{array}$$

where *M*_0_ and *M*_1_ represent sample weight before and after immersion, respectively.

### 2.9. Mechanical Properties

Tensile strength and elongation at break of the composite films were evaluated using a universal testing machine (AGS-J 1 kN, Japan) according to the ASTM D882-02 standard method. Briefly, the films were cut into rectangular shapes (10 × 50 mm) and kept at 25°C with relative humidity of 50% ± 2% until reaching constant weight. The films were tightly fixed in the grips with 30 mm initial space and pulled apart by a 1 kN load cell. The experiment was conducted in triplicate. The tensile strength and elongation at break were calculated from the following equations, respectively [57]:

(4) $$\begin{array}{c} tensile\;strength=\frac{F}{\left(T\;x\;W\right)}\text{,} \end{array}$$

where *F*, *T*, and *X* are the maximum force, film thickness, and width of film, respectively.

(5) $$\begin{array}{c} Elongation\;at\;break=\left(D_{1}-D_{0}\right)\;x\;100\text{,} \end{array}$$

where *D*_1_ is the distance of rupture and *D*_0_ is the initial distance between grips.

### 2.10. Cytotoxicity

Cytotoxicity test was conducted using the colorimetric assay which is based on the ability of the cells (cellular oxidoreductase enzymes) to reduce the tetrazolium dye 3-(4,5-dimethylthiazol-2-yl)-2,5-diphenyltetrazolium bromide (MTT) to its insoluble formazan, following the ISO 10993-5 standard [59]. Mouse fibroblast cells L929 (NCTC clone 929 : CCL1 from the American Type Culture Collection (ATCC), strain L) were cultured in minimum essential medium (MEM) with an appropriate cell density of 10^5^ cells/ml and incubated for 24 ± 2 h. The films were sterilized by UV irritation for 15 min, prepared at 3 cm^2^/ml, and extracted for 24 ± 2 h at 37 ± 1°C. The extracts were further incubated for 24 ± 2 h before being stained with MTT assay for 2 h. A Thermanox (Nunc) coverslip was used as the negative control, while polyurethane film containing 0.1% zinc diethyldithiocarbamate served as the positive control. After culturing with fibroblast cells for 24 h, tested samples were compared to the negative and positive controls. A microplate reader was used to determine cell viability at absorbance of 570 nm using equation (6) [25]. Cell viability above 70% from equation (6) was considered noncytotoxic.

(6) $$\begin{array}{c} Cell\;viability=\frac{{OD}_{sample}}{{OD}_{blank}}\;x\;100\%\text{,} \end{array}$$

where OD_sample_ and OD_blank_ are measurements of optical density of the test sample and blank sample, respectively.

### 2.11. Antimicrobial Ability

Antimicrobial property of the films loaded with the crude betel leaf extract was studied against *Staphylococcus aureus* DMST 8840 and *Pseudomonas aeruginosa* TISTR 781 using the agar diffusion method. Before the experiment, *S. aureus* and *P. aeruginosa* suspensions of 10^6^ CFU/ml were evenly spread over nutrient agar plates. Films loaded with the betel leaf extract were placed on an inoculated agar surface, with paper discs loaded with erythromycin used as the positive control and PG as the negative control. The culture plates were incubated at 37°C for 18 h, and the inhibition zone was measured at the end of the incubation time using digital Vernier caliper [60].

### 2.12. Statistical Analysis

The statistical analysis of data was done by one-way analysis of variance (ANOVA) at a confidence level of 0.05 using the Minitab program. All data were expressed as mean ± standard deviation. A *p* value <0.05 was considered statistically significant.

## 3. Results and Discussion

### 3.1. Characterization of Nanocellulose

**Figure 1:**
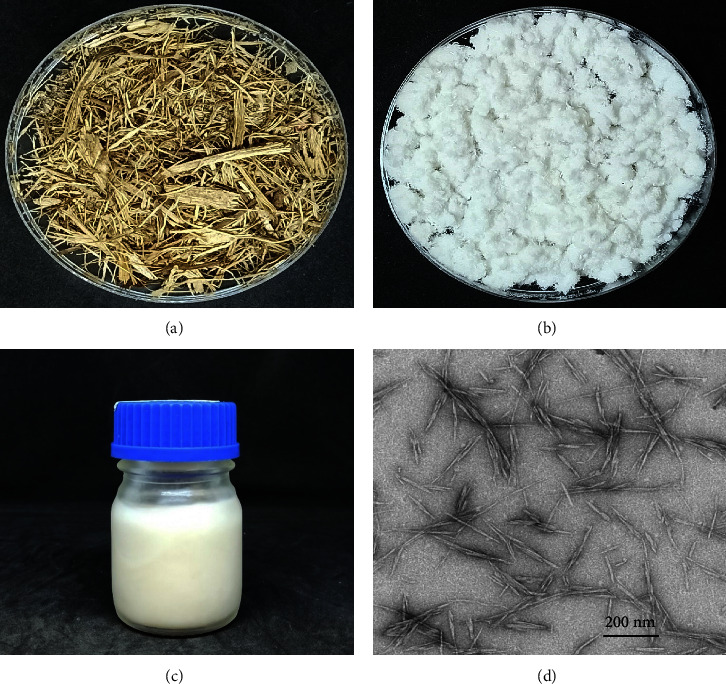
Photographs of (a) SCB, (b) bleached fiber, (c) CNCs, and (d) TEM image of CNCs.

SCB (Figure 1(a)) consists of cellulosic and noncellulosic components. A series of treatment steps are involved in removing the noncellulosic components (hemicellulose and lignin) to promote defibrillation. Removal of major noncellulosic components increased the cellulose content (Figure 1(b)). The bleached fiber was subsequently subjected to acid hydrolysis that caused disordering in the glycosidic linkages and breakdown of the fibrous structures. These resulted in the reduction of fiber size to produce CNCs. The crystalline CNCs were presented as a stable colloidal suspension (Figure 1(c)). The surface of CNCs was linked to negatively charged particles of sulfate half ester groups derived from the acid hydrolysis process [35]. TEM analysis was carried out to determine particle size and distribution of CNCs. Results showed needle-like shapes of individual and aggregated particles (Figure 1(d)). Dimensions of the CNCs were approximately 104 nm in length and 7 nm in width, similar to sizes reported earlier [61, 62]. The stable colloidal property coupled with nanosize of particles make the CNCs an ideal candidate in composite film preparation.

### 3.2. Characterization of Composite Film

#### 3.2.1. Optical Observation

**Figure 2:**
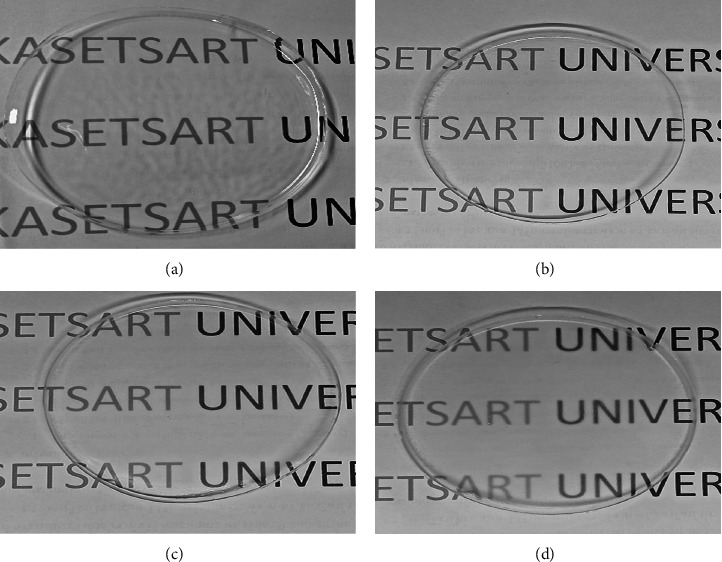
Photographs of (a) PVA, (b) PVA/GT, (c) PVA/GT/CNC2, and (d) PVA/GT/CNC10.

Table 1 shows the percentage transparency of the composite films. Neat PVA recorded the highest transmittance of 91.27 ± 0.43% followed by PVA/GT at 74.59 ± 0.43%. However, the incorporation of CNCs has drastically reduced the transparency of films. The PVA/GT/CNC10 film recorded the lowest transmittance of 46.28 ± 0.54%. High significant difference (*p* < 0.05) was recorded among PVA, PVA/GT, and PVA/GT/CNC composite films due to differences in light dispersion caused by the disparate viscosities of GT and PVA [63] and aggregation of CNCs as the concentration increased [64]. Optical properties also relate to the rearrangement in the internal structure of PVA molecules during the drying process [63, 65]. Although the opaqueness of films increased at higher CNC concentration (Table 1), visual observation showed they are all colorless and transparent (Figure 2). The film is ideal for wound dressings, as it will allow daily observation without its removal and prevents damage to the wound bed [49]. Optically transparent wound dressings offer an opportunity to monitor the wound healing progress without having to replace the dressing. In addition, the direct naked-eye observation is the most appropriate and effective way for detecting wound infection during the healing process [66, 67]. Films with the lowest and highest concentrations of CNCs were, therefore, selected for SEM analysis.

#### 3.2.2. SEM

**Figure 3:**
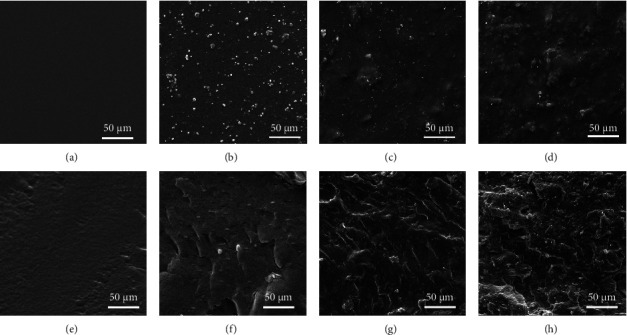
SEM micrographs of surface of (a) PVA, (b) PVA/GT, (c) PVA/GT/CNC2, and (d) PVA/GT/CNC10, and cross section of (e) PVA, (f) PVA/GT, (g) PVA/GT/CNC2, and (h) PVA/GT/CNC10 composite films.

SEM micrograph (Figure 3) shows the surface and cross section of neat PVA, PVA/GT, and PVA/GT/CNC films. The neat PVA film had a uniform texture with a smooth planer surface and cross section (Figures 3(a) and 3(e)). However, the addition of GT changed the film surface to slightly rough, characterized by the presence of white dots on the surface and cracks in the cross section (Figures 3(b) and 3(f)). Mostafavi et al. [57] reported that the chemical structure of GT could organize into a more open and porous network [47]. Similarly, the film containing GT showed reduced homogeneous quality [65, 68]. Dispersion of CNCs in the PVA/GT films (Figure 3(c)) resulted in the disappearance of some white dots on the surface structure, suggesting that CNC particles filled in the polymeric matrix. Increasing the CNC content led to assemblage and cluster formation of CNCs in the film (Figure 3(d)). This result agreed with a previous study by Jahan et al. [69]. The CNC particles were observed on the fractured shapes in the cross section (Figures 3(g) and 3(h)). The nanocellulose reinforced polymer network formed aggregates with a wide range of sizes and shapes in random directions. However, higher concentrations of nanocellulose induced brittle fracture due to the aggregation at localized points.

#### 3.2.3. FTIR Analysis

**Figure 4:**
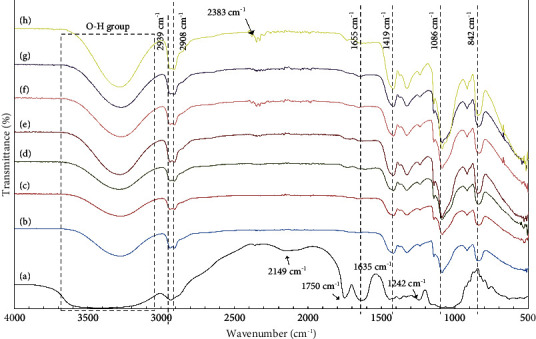
FTIR spectra of (a) GT, (b) PVA, (c) PVA/GT, (d) PVA/GT/CNC2, (e) PVA/GT/CNC4, (f) PVA/GT/CNC6, (g) PVA/GT/CNC8, and (h) PVA/GT/CNC10 composite films.

Figure 4 shows the FTIR analysis of chemical functional groups of GT powder and PVA, PVA/GT, and PVA/GT/CNC films. The FTIR spectra for GT powder with an absorption band at 2149 cm^−1^ corresponded to various carbonyl groups in the gum while peaks of carbonyl stretching in aldehydes, ketones, and carboxylic acids were presented at 1750 cm^−1^ [70]. The bands at 1635 cm^−1^ and 1442 cm^−1^ were attributed to asymmetrical and symmetrical stretching of carboxylate groups, respectively, while peaks at 1242 cm^−1^ and 1020 cm^−1^ displayed C-O stretching vibration in polyols and alcoholic groups, respectively [22, 24, 25]. The band observed in all samples at 3285 cm^−1^ was characteristic of O-H stretching groups from intra- and intermolecular hydrogen bonds [15, 69] while a wider band of O-H stretching in the GT structure observed at 3420 cm^−1^ was caused by OH and COOH groups [25, 57]. Asymmetrical and symmetrical stretching vibrations of methylene groups were presented at 2939 cm^−1^ and 2908 cm^−1^, respectively [24], while the peak at 1086 cm^−1^ was assigned to C-O stretching [57, 69]. Vinyl C-H in plane bending of PVA was confirmed at 1419 cm^−1^. Furthermore, the absorption band centered at 842 cm^−1^ represented C=C bending [71, 72]. No significant changes were observed in the chemical structure of PVA after the incorporation of GT and CNCs. Notably, addition of CNCs at high concentration contributed C=C stretching at 1655 cm^−1^ [73].

#### 3.2.4. Swelling Ratio of Composite Film

**Figure 5:**
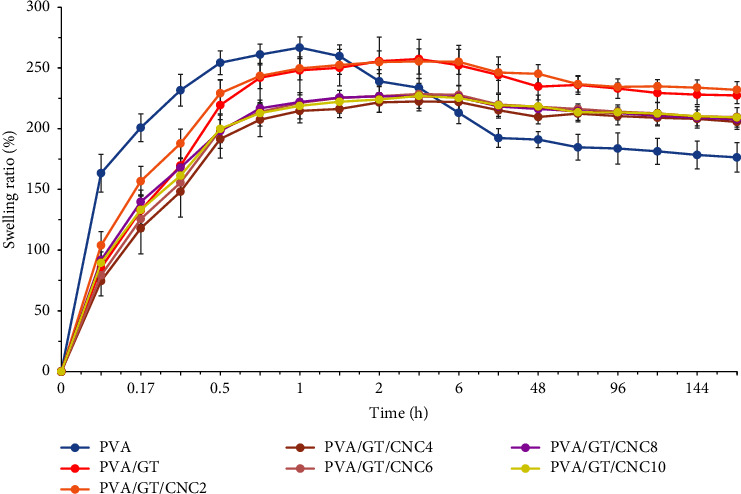
Swelling ratio of neat PVA, PVA/GT, and PVA/GT/CNC composite films.

Results in Figure 5 show the swelling ability of the various films. At the initial stage, the large number of free hydroxyl groups in the PVA film absorbed water molecules to reach a maximum state of approximately 260% before reducing at 1.5 h. PVA/GT films increased gradually and reached a steady rate of 250% due to the presence of hydrogen bonds in hydroxyl and carboxyl functional groups [70]. The porous structure of GT helped in trapping water molecules [20, 68, 74]. After 6 h, swelling ability slightly decreased to 230% for 7 days due to degradations in the polymer matrix [75]. There were no obvious differences in PVA/GT/CNC2 and PVA/GT films. Addition of small amounts of CNCs increased water absorption in the polymer matrix. However, as CNC concentration increased, extra swelling capacity was restrained [58, 69, 76] due to the reinforcing effects of CNCs. This reduction illustrated the hydrogen bonding between CNCs and polymers. Thus, water molecules could not freely pass through the polymer [76, 77]. The PVA/GT/CNC2 film provided swelling behavior that could create an environment suitable for wound healing [14, 17, 78, 79]. Swelling of wound dressing is an important factor that relates to wound exudate absorption and prevention of infection. This is as a result of physical and chemical changes in material structure that help water molecules to diffuse internally, leading to an increase in free volume [69, 78, 80].

#### 3.2.5. Stability

**Figure 6:**
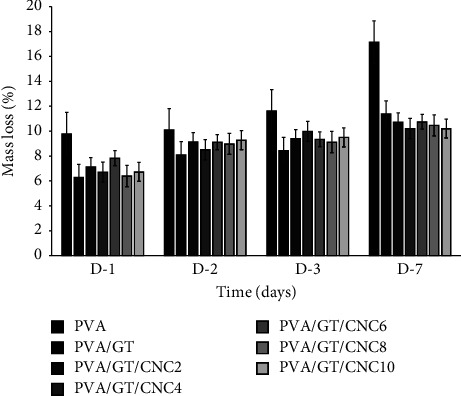
Percentage weight loss of PVA, PVA/GT, and PVA/GT/CNC composite films.

Stability of the wound dressing was assessed as the percentage weight loss of composite film over a period of 7 days to indicate its prolonged use. The result in Figure 6 shows that the PVA film exhibited the highest rate of weight loss of 9.8% on the first day, which increased to 17.2% on the 7^th^ day due to solvation and fragmentation of the film [80]. There was significant difference between the neat PVA and the composite films. The addition of GT improved the film stability by maintaining weight loss below 12% for 7 days. Bassorin fragments in GT are insoluble in water; these probably reduced the film's solubility [81]. The solubility was further reduced when CNCs were added. This could be explained as due to the formation of a strong matrix of hydrogen bonds through the three-dimensional structure of CNCs and the polymeric matrix that reduced free hydroxyl groups and restricted water penetration [58, 77].

#### 3.2.6. Mechanical Properties

**Figure 7:**
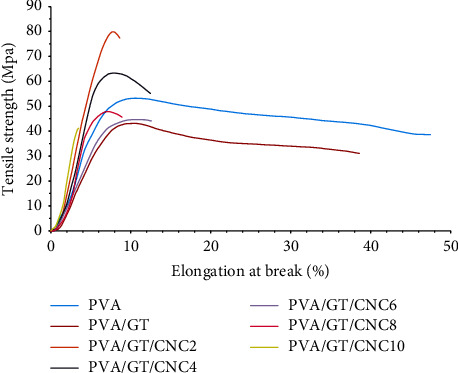
Stress-strain curves of PVA, PVA/GT, and PVA/GT/CNC with different concentrations of CNC (2 to 10%).

Mechanical properties of the films are summarized in Table 2. Wound dressing should be strong, flexible, and elastic for efficient treatment; thus, the film was evaluated in terms of tensile strength, elongation at break, and elastic modulus. The neat PVA film exhibited a tensile strength of 54.63 MPa, which reduced slightly to 49.26 MPa when GT was added. There was no significant difference (*p* < 0.05) between neat PVA and PVA/GT. This result agreed with Ojagh et al. [82] who found that the addition of GT had no significant effect on mechanical properties. Highest tensile strength was recorded in PVA/GT/CNC2 (80.39 MPa), while PVA/GT/CNC6 gave the lowest strength of 45.05 ± 3.39 MPa. The addition of CNCs increased the strength of the material by entrapping inside the matrix. This allowed strong hydrogen bond formation between the nanocellulose and PVA matrix, thus impacting mechanical integrity [61, 83]. However, high concentration of CNCs led to agglomeration of particles, increased rigidity, and poor distribution in polymer matrices, which affected the formation of hydrogen bonds among polymer chains and inhibited reinforcing properties [15, 71]. Elastic modulus of PVA and PVA/GT was 1223.08 ± 182.08 MPa and 1062.51 ± 101.65 MPa, respectively. Highest elastic modulus of 1526.11 ± 31.86 MPa was recorded in the PVA/GT/CNC2 film, which decreased to 1260.45 ± 76.94 MPa in PVA/GT/CNC10. The high value was attributed to the crystalline nature of CNCs that resulted in better alignment and enhanced elastic modulus [69, 83]. There was no significant difference in elastic modulus of neat PVA and composite films loaded with CNCs, which implies the nanoparticles has no negative effect on the elastic nature of PVA. Neat PVA had elongation at break of 48.52% and this slightly decreased to 44.48% in PVA/GT. However, elongation at break of PVA/GT/CNC films decreased drastically with increasing concentration of CNCs. The elongation at break significantly decreased to 8.11% in PVA/GT/CNC2 and dropped further to 3.92% in the PVA/GT/CNC10 film due to the rigid nature of CNCs. Since CNCs are nondeformable, strong interaction between CNCs and the polymer matrix did not allow elongation in the composite materials [84]. The tensile strength of wound dressings should be adequate for application and storage to ensure that it is not easily damaged by handling [85, 86]. Therefore, PVA/GT/CNC composite materials in the range of 45–80 MPa showed good mechanical properties compared to previous studies [86–88] for wound dressing. The stress-strain curves of PVA, PVA/GT, and PVA/GT/CNC with different concentrations of CNC are presented in Figure 7.

#### 3.2.7. Cytotoxicity

**Figure 8:**
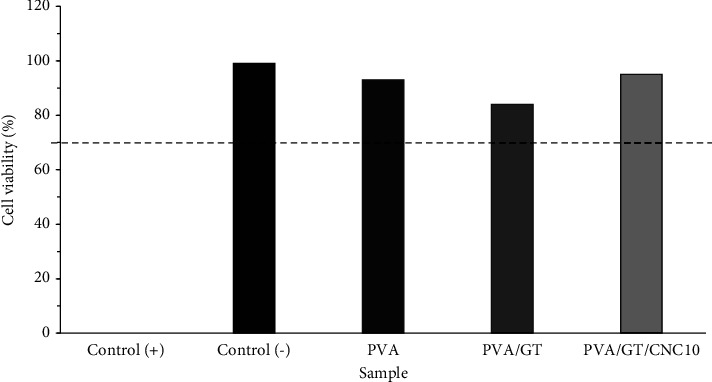
Cell viability of PVA, PVA/GT, and PVA/GT/CNC10 compared with positive and negative controls.

The cytotoxicity of PVA, PVA/GT, and PVA/GT/CNC10 films was evaluated using the MTT assay. The sample with the highest CNC concentration (PVA/GT/CNC10) was tested to confirm nontoxicity and safety of the film for cell growth. The result in Figure 8 shows all the test samples except that the positive controls (which is expected) are nontoxic to fibroblast cells with reference to ISO 10993-5 standard with acceptable limit of 70% cell viability [59, 89]. Cell viability of PVA and PVA/GT was 93% and 84%, respectively. The decrease in cell viability of the PVA/GT film may be due to the diversity in chemical composition that slightly influenced the biological properties of the film [20]. The PVA/GT/CNC10 film, which contains high concentration of CNCs, was found to be nontoxic to fibroblast cells with 95% cell viability. The value is closer to that of the negative control of 99% cell viability. Incorporation of CNCs in the polymeric matrix and reaction between CNCs and PVA/GT improved cell viability in the films. The film satisfied noncytotoxic requirement of cell viability above 70% in agreement with previous reports [24, 89, 90].

#### 3.2.8. Antibacterial Activity

**Figure 9:**
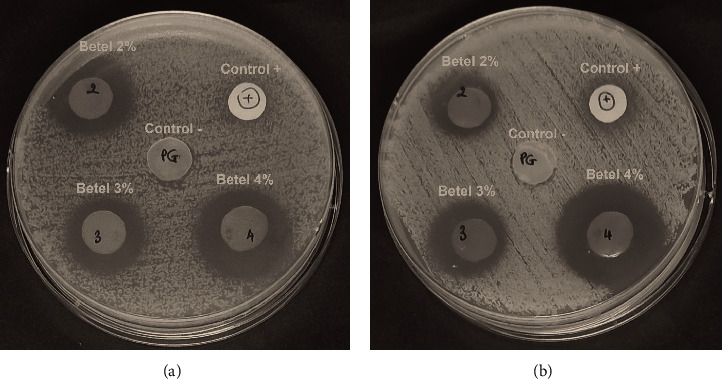
Antibacterial activity of PVA/GT/CNC2 films loaded with 2%, 3%, and 4% of crude betel extracts against *P. aeruginosa* (a) and (b) *S. aureus*.

Figure 9 shows clear zones around discs, which increased with increasing concentration of the betel leaf extract. Disc diffusion result revealed that films loaded with the betel leaf extract at different concentrations of 2%, 3%, and 4%, exhibited excellent antibacterial activity against both gram-negative (*P. aeruginosa*) and gram-positive (*S. aureus*) bacteria. There was high significant difference in inhibition zone between the positive control (erythromycin) and the films loaded with extracts (Table 3). As expected, there was no inhibition zone in the negative control. The film loaded with 4% extract (PVA/GT/CNC2_4%) recorded the highest inhibition zone of 23.60 ± 0.55 mm and 28.20 ± 0.84 mm for *P. aeruginosa* (Figure 9(a)) and *S. aureus* (Figure 9(b)), respectively. In all, *S. aureus* is more susceptible than *P. aeruginosa* which may be as a result of the differences in their cell wall structure [91, 92]. The cell wall of *S. aureus* has only peptidoglycan layer, while that of *P. aeruginosa* is made of three layers such as the inner or cytoplasmic membrane, peptidoglycan layer, and outer membrane [91, 93, 94]. The betel leaf extract contained an essential oil and phenolic compounds which are reported to inhibit bacterial cell growth [95–97]. The presence of oxygenated terpenoids including alcohols and phenolic terpenes [96] as well as hydroxyl groups in hydrophobic fatty acids and fatty acid ester components caused destabilization of the cytoplasmic membrane, disrupted proton and electron flow, and decreased adenosine triphosphate (ATP) synthesis, leading to cell death [44, 98].

## 4. Conclusions

The PVA/GT/CNC composite film was successfully prepared as wound dressing material. The films exhibited higher swelling ratio and mechanical properties with good transparency, which are ideal for wound dressing. These properties could help to absorb exudates with easy daily observation and minimize trauma to the wound bed. Incorporation of CNCs from 2% to 10% improved the physicochemical properties of the film. The PVA/GT/CNC2 showed excellent properties among the CNC-based composite films. Apart from the physicochemical enhancements, the cytocompatibility was also improved. The film loaded with the betel leaf extract exhibited excellent antibacterial activity against *P. aeruginosa* and *S. aureus* strains. Therefore, the prepared nanocomposite film with good features will provide the optimum conditions for wound healing, especially in cutaneous wound dressing application. The visual inspection property of PVA/GT/CNC coupled with antibacterial activity from the betel leaf extract and other excellent physiochemical characteristics of the film are suitable for wound healing application. This dressing can protect the wound surface from infection and dehydration, which deprives wound tissues of oxygen and the nutrients necessary for healing.

## Figures and Tables

**Table 1:** Transparency of PVA/GT composite films with different concentrations of CNCs.

Samples	Transmittance
PVA	91.27 ± 0.43^b^
PVA/GT	74.59 ± 0.43^c^
PVA/GT/CNC2	74.30 ± 0.17^c^
PVA/GT/CNC4	68.94 ± 0.11^d^
PVA/GT/CNC6	68.79 ± 0.11^d^
PVA/GT/CNC8	59.70 ± 0.39^e^
PVA/GT/CNC10	46.28 ± 0.54^f^
Control (blank)	100 ± 0.00^a^

For values with the same letter, the difference is not statistically significant, while different letters mean statistical significance (*p* ≤ 0.05).

**Table 2:** Mechanical properties of PVA/GT/CNC composite films.

Samples	Tensile strength (MPa)	Elongation at break (%)	Elastic modulus (MPa)
PVA	54.63 ± 0.69^bc^	48.52 ± 1.57^a^	1223.08 ± 182.08^a^
PVA/GT	49.26 ± 5.00^bc^	44.38 ± 5.03^a^	1062.51 ± 101.65^b^
PVA/GT/CNC2	80.39 ± 1.41^a^	8.11 ± 0.30^b^	1526.11 ± 31.86^a^
PVA/GT/CNC4	59.09 ± 4.26^b^	7.36 ± 0.82^b^	1450.74 ± 76.94^a^
PVA/GT/CNC6	45.05 ± 3.39^c^	8.81 ± 1.56^b^	1299.13 ± 120.37^a^
PVA/GT/CNC8	47.63 ± 3.54^bc^	6.88 ± 0.06^b^	1353.38 ± 177.19^a^
PVA/GT/CNC10	47.68 ± 9.26^bc^	3.92 ± 0.92^b^	1260.45 ± 76.94^a^

For values with the same letter, the difference is not statistically significant, while different letters mean statistical significance (*p* ≤ 0.05).

**Table 3:** Antimicrobial activity of PVA/GT/CNC2 film loaded with betel extract on the agar diffusion test.

Samples	Diameter of inhibition zone (mm)	
	*P. aeruginosa*	*S. aureus*
PVA/GT/CNC2_2%	16.60 ± 1.03^b^	18.00 ± 0.79^b^
PVA/GT/CNC2_3%	19.00 ± 1.41^b^	22.00 ± 1.13^b^
PVA/GT/CNC2_4%	23.60 ± 0.55^a^	28.20 ± 0.84^a^
Positive control	12.30 ± 0.57^c^	14.40 ± 1.14^c^
Negative control	—	—

For values with the same letter, the difference is not statistically significant, while different letters mean statistical significance (*p* ≤ 0.05).

## Data Availability

The data that support the findings of this study are available upon reasonable request from the corresponding author.
